# Hypertrophied medial parapatellar plica: a case of a medial plica anatomical variation with insertion to the inter-meniscal ligament in an adolescent athlete treated arthroscopically

**DOI:** 10.1007/s00276-024-03338-5

**Published:** 2024-03-09

**Authors:** Angelo V. Vasiliadis, Nikolaos E. Koukoulias, Theofilos Dimitriadis, Trifon Totlis

**Affiliations:** 1 Department of Orthopaedic Surgery, Sports Trauma Unit, St. Luke’s Hospital, Panorama, Thessaloniki, 55236 Greece; 2 Department of Anatomy and Surgical Anatomy, Faculty of Health Sciences, School of Medicine, Aristotle University of Thessaloniki, Thessaloniki, Greece

**Keywords:** Medial parapatellar plica, plica syndrome, Arthroscopy, Knee anatomy

## Abstract

**Purpose:**

The present study aims to report the arthroscopic, radiological and clinical appearance of a rare anatomical variation of a hypertrophied medial parapatellar plica with its response to arthroscopic treatment.

**Case presentation:**

A 14-year-old female handball athlete presented with a history of left knee injury during her participation in a handball training session and subsequent locked knee at 20º flexion. Tenderness was located at the medial joint line. Plain radiographs of the injured knee were normal. The magnetic resonance imaging revealed a hypertrophic medial parapatellar plica and a horizontal tear of the medial meniscus. A standard knee arthroscopy was performed. An extremely hypertrophied medial plica was identified, covering a great part of the medial femoral condyle extending up to the femoral trochlea. Distally, it was attached into the inter-meniscal ligament. The plica was excised and the medial meniscus tear was repaired. At 1-month post-operatively, the patient was completely asymptomatic and at 3-months she returned to her weekly training routine.

**Conclusions:**

This study presented a rare anatomical variation of a hypertrophied medial parapatellar plica with atypical course in the medial patellofemoral compartment and insertion into the inter-meniscal ligament. In combination with a medial meniscus tear led to a locked knee. Arthroscopic medial meniscus repair and plica excision resulted in complete resolution of symptoms.

## Introduction

**Fig. 1:**
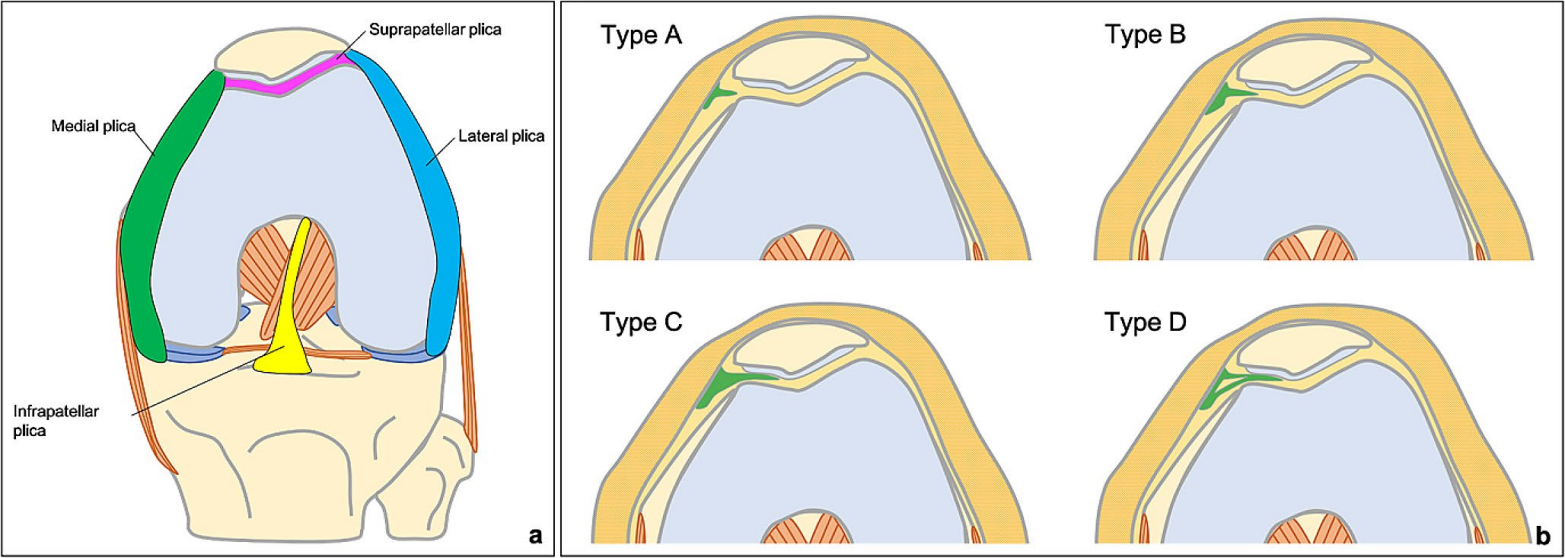
Schematic illustration showing the common plicae in the knee, anterior view with knee slightly flexed, displaying suprapatellar, infrapatellar, medial and lateral plicae (**a**) and the classification of medial plicae according to Sakakibara (**b**)

Synovial plicae in the knee joint are inward folds of the synovial lining, which are present during embryological development and are classified anatomically as suprapatellar, infrapatellar, medial and lateral plicae (Fig. 1a) [5]. The medial plica is present in up to 50% of the individuals [2] and is classified by Sakakibara based on its appearance in arthroscopy as type A (cord-like), type B (shelf-like), type C (shelf-like, covering the medial femoral condyle) and type D (with double insertion) (Fig. 1b) [8]. Mechanical irritation to the knee joint may cause medial plica to become inflamed and thickened, leading the plica to snap over the medial femoral condyle and to cause synovitis, pain and chondral damage [2]. This situation is referred to as medial plica syndrome and it is difficult to diagnose, because its symptoms mimic meniscus tear, patellofemoral syndrome or osteochondritis dissecans symptoms [1].

Normally, medial plica arises on the medial wall of the synovial pouch, under the medial retinaculum or to the genu articularis muscle, while courses in a coronal plane parallel to the medial edge of the patella and medial femoral condyle. The medial plica attaches distally on the infrapatellar fat pad or medial patellotibial ligament [1, 3]. In the present study, we report the arthroscopic, radiological and clinical appearance of a rare anatomical variation of the medial plica along with its response to arthroscopic treatment.

## Case presentation

A 14-year-old female handball athlete (160 cm; 52 kg) presented with a history of left knee injury during her participation in a handball training session. She had no history of past musculoskeletal surgery or any other illness and no relevant family background. The patient had been playing handball (position: goalkeeper) for 3 years. On physical examination, the patient presented with knee locked at 20º and tenderness at the medial joint line. Her gait was slightly painful with no full range of motion (ROM). Clinical tests were not applicable due to the locked knee. The neurovascular examination was normal.

**Fig. 2:**
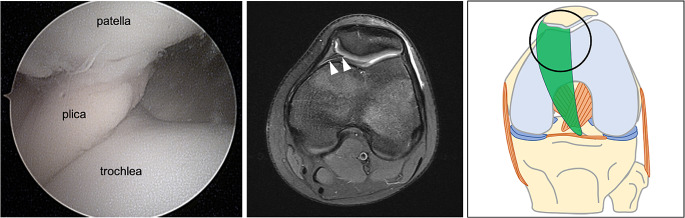
Arthroscopic and magnetic resonance imaging (MRI) image of the anatomic variation of the medial plica (white arrowhead). The patella can be seen in front of the femoral trochlea, with a hypertrophied plica interposed. In the drawing, the circle demonstrates the position of the knee arthroscopy and MRI image

**Fig. 3:**
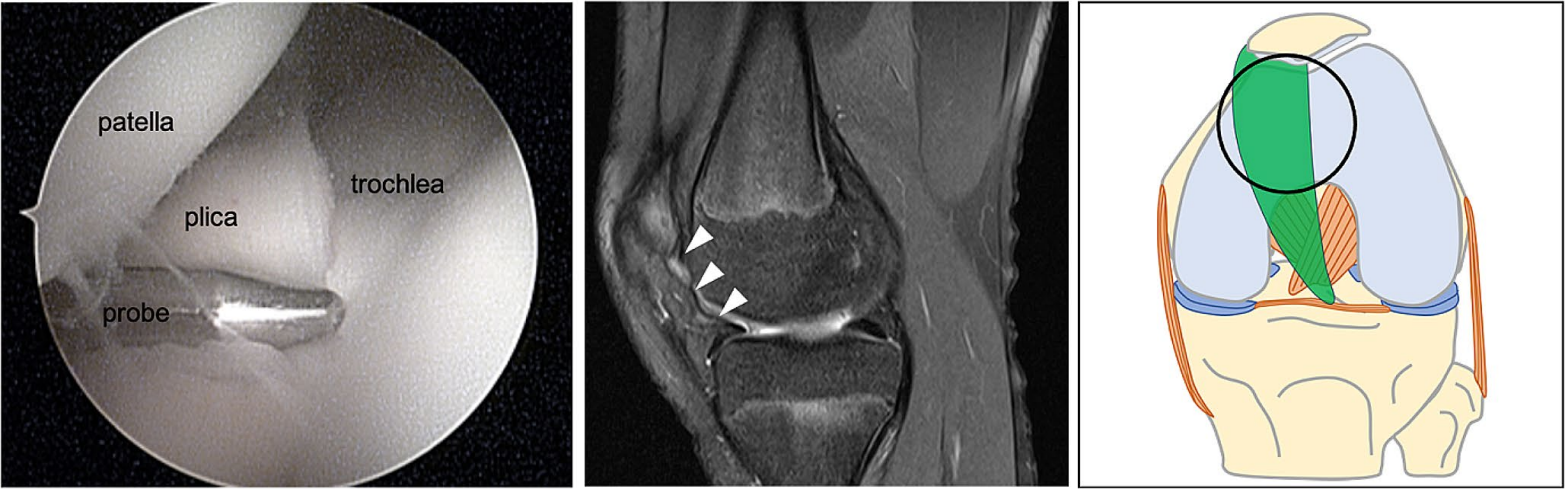
Arthroscopic and magnetic resonance imaging (MRI) image of the anatomic variation of the medial plica (white arrowhead). The hypertrophied plica can be seen interposed between the patella and the femoral trochlea, while it is slightly retracted with the probe (cover most of the trochlea groove). In the drawing, the circle demonstrates the position of the knee arthroscopy and MRI image

**Fig. 4:**
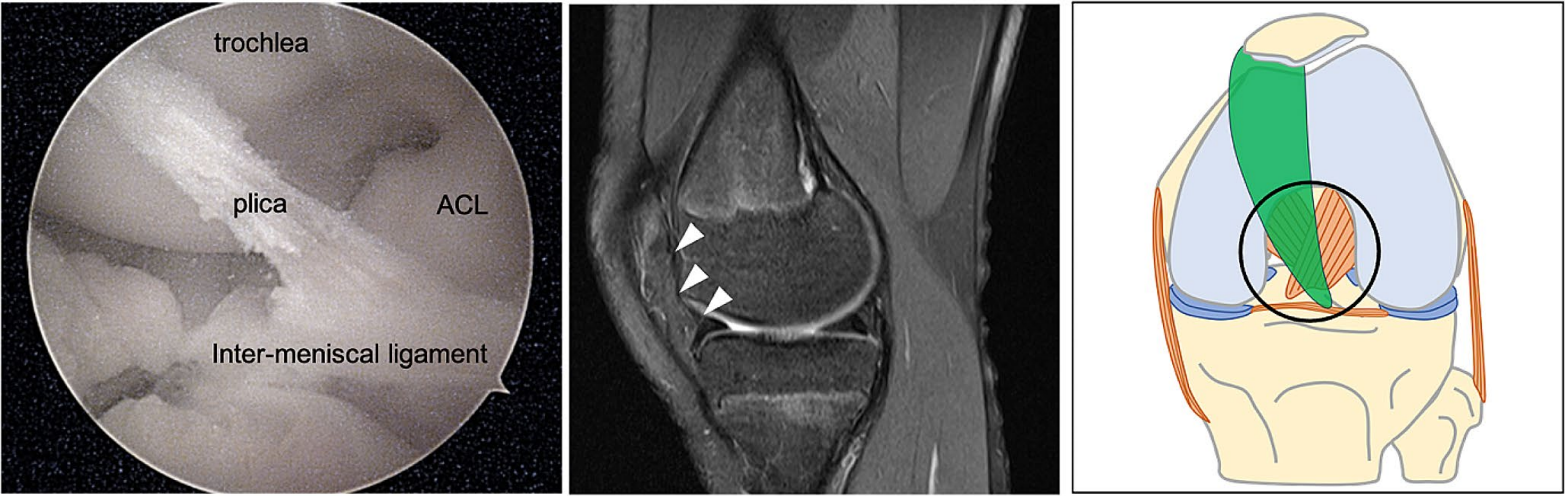
Arthroscopic and magnetic resonance imaging (MRI) image of the anatomic variation of the medial plica (white arrowhead). After partial debridement, the shaver is placed in the distal part of its attachment in the inter-meniscal ligament in front of the anterior cruciate ligament (ACL). In the drawing, the circle demonstrates the position of the knee arthroscopy and MRI image

The radiographs were normal and she underwent a magnetic resonance imaging (MRI) (Siemens MAGNETOM Avanto Fit with BioMatrix technology, 1.5 Tesla) in a supine position with her knee in 20 degrees of flexion. Intravenous contrast was not administered. The MRI revealed a hypertrophic medial parapatellar plica (Figs. 2, 3 and 4) and a horizontal tear of the medial meniscus. Based on the clinical image and MRI findings, knee arthroscopy was recommended.

**Fig. 5:**
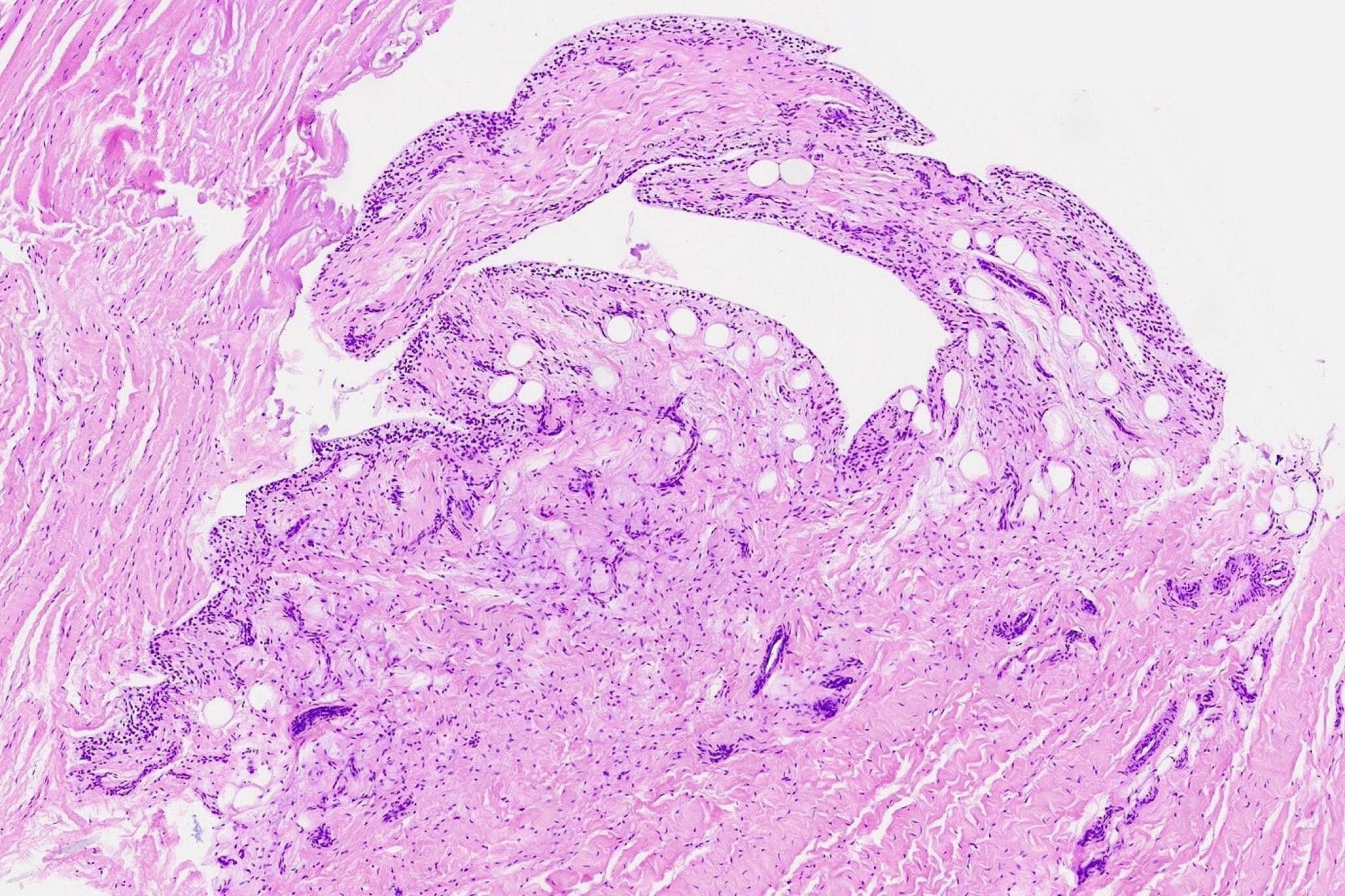
Histological findings of the resected medial parapatellar plica. Fibroblastic tissue is identified consisting of spindle cells with mild vascularization, covered on its surface by synovial membrane cells, which in turn shows mild hyperplastic changes with slightly papillary architecture in places. Development of mild to moderate of inflammatory cells was observed in fibrous tissues covered with synovial membrane cells

A standard knee arthroscopy was performed under general anaesthesia. An extremely hypertrophied medial parapatellar plica was identified using the arthroscopic probe. The plica was covering a great part of the medial femoral condyle extending up to the femoral trochlea. Distally, it was attached into the inter-meniscal ligament (Fig. 3). The plica was completely excised from its proximal attachment on the medial wall of the synovial pouch to its distal insertion using a mechanical shaver. The medial meniscus tear was repaired using an all-inside suture meniscal repair device (Fast-Fix, Smith & Nephew, USA). No other intra-articular pathology was found. The patient was discharged on the same day and allowed full weight-bearing as tolerated with arm crutches. Histological findings revealed fibrous and inflammation characteristics, covered with synovial membrane cells (Fig. 5). At 1-month follow-up, the patient was completely asymptomatic and at 3-months follow-up, she has returned to her weekly training routine.

## Discussion

Synovial plicae are normal folds within knee joint, which are present in about 50% of individuals [5]. The medial parapatellar plica is common to cause mechanical symptoms, depending on its position, size and elasticity [6]. The embryological development of the knee plicae is still controversial [2, 6]. An old theory describes the existence of a cartilaginous centre, which will be developed the articular cavity of the knee [4]. In this theory, the centre has three layers, two cartilaginous and one intermediate layer. The development of the intermediate centre is complex, creating the medial and inferior plicae, as well as the meniscus and cruciate ligaments [4]. However, the most common theory support that the formation of knee joint started as multiple cavities, patellofemoral, femoromeniscal and meniscotibial, in the 8th week of embryogenesis, with subsequent fusion of those cavities by the end of 12th week. Any incomplete resorption of those mesenchymal cavitations result in the knee plicae [6].

Multiple proximal attachments have been described in the literature for the medial parapatellar plica. The most common insertion is in the medial wall of the synovial pouch, under the medial retinaculum or to the genu articularis muscle. However, according to the literature the distal attachment is constant at the infrapatellar fat pad [1, 3] or may be continuous with the anterior margin of an infrapatellar plica [9]. From a phylogenetic point of view, infrapatellar plica is a remnant between the femoro-tibial and femorofibula articulations, which is present in reptiles and amphibians [9]. In the present case, the hypertrophied medial parapatellar plica typically arised from the medial wall of the synovial pouch, but it was extremely hypertrophied in the medial patellofemoral compartment and presented an atypical distal insertion into the inter-meniscal ligament. This anatomical variation could be characterized a combination of medial and infrapatellar plica, with alterations in separation and resorption during embryonic development. The origin of the infrapatellar plica is usually from the intercondylar notch in the femoral footprint of the anterior cruciate ligament and attaches onto the synovium around the infrapatellar fat pad [9].

Histologically, normal plicae are characterized by a lining of synovial cells, while pathologic plicae frequently show increased vascularity and appearance of inflammatory cells [9]. However, in the current case, histological findings revealed a lining of single or reduplicated synovial cells, the specific characteristics of a normal plica, on a stroma of generalized fibrosis, as well as inflammatory cells into the fibrous tissues indicating the sub-acute phase of the reaction.

Occasionally, medial plica becomes symptomatic, as a result of overuse or history of injury [5]. This can be caused during repetitive knee motion, between 30º to 60º of flexion, with an engagement of the plica to the medial femoral condyle [2]. Despite the fact that this condition can cause anteromedial patellofemoral cartilage damage in up to 80% of the patients [2], the present case report did not reveal any cartilage involvement maybe due to the young age of the patient and the more elasticity of the tissues. Some patients may present with a sensation of mild medial or/and anterior knee pain, while others may exhibit crepitus, snapping or popping, effusion and joint locking [2, 5, 7]. In our case, anterior knee pain and locking knee was the predominant symptoms, due to a twisting injury during handball training session.

In addition, it is crucial to differentiate medial plica syndrome symptoms from other pathologies mimicking this condition, such as meniscus tear and patellofemoral syndrome-like symptoms [2, 9]. The diagnosis of medial plica syndrome is therefore based on non-specific clinical symptoms, MRI findings and clinical suspicion. Also, MRI can determine if there are multiple pathologies that can contribute to this condition, while can help guide the diagnosis with a sensitivity and specificity of 93% and 81%, respectively [2]. In the current case, both a hypertrophied anatomic variation of medial parapatellar plica and a horizontal medial meniscus tear was present. Therefore, the primary source of symptoms was not clear. However, the uncommon hypertrophy of the plica along with its atypical course and distal attachment is a rare anatomical variant. Apart from the anatomical interest, radiologists and orthopaedic surgeons should be aware of this anatomical variation as it may be found in a knee MRI or arthroscopy.

Initial treatment management for the medial parapatellar plica is conservative approach, including rest, cryotherapy, anti-inflammatory medication, physiotherapy and modification of activities of daily living [2]. However, surgical treatment with arthroscopic excision should be considered after failed conservative treatment, when pain persists over a long period [2], or/and when the medial plica covers part of medial femoral condyle [5]. In such cases, surgical treatment reduces the potential for erosion of the cartilage surface [3]. In the present case, immense arthroscopic treatment was applied based on both clinical appearance and MRI findings of the patient. Post-operatively, complete resolution of symptoms and resume of daily and sports activities was achieved.

## Conclusions

A rare anatomical variant of an extremely hypertrophied medial parapatellar plica with atypical course in the medial patellofemoral compartment and insertion into the inter-meniscal ligament was reported in an adolescent athlete. The plica associated with a horizontal meniscus tear caused knee locking and arthroscopic treatment was needed. Plica resection and meniscus repair resulted in complete resolution of symptoms and return to activities.

## Data Availability

The data and materials used in this study are available from the corresponding author upon reasonable request (Trifon Totlis).
